# Automatic determination of glymphatic flow with the DTI‐ALPS‐index along the principal axis system in native imaging space corrects for head and fiber orientation

**DOI:** 10.1002/mrm.70082

**Published:** 2025-10-05

**Authors:** Ali Ajouz, Olav Jansen, Lynn Johann Frohwein, Svea Seehafer, Naomi Larsen, Jan‐Bernd Hövener

**Affiliations:** ^1^ Department of Radiology and Neuroradiology UKSH, CAU Kiel Kiel Germany; ^2^ Section Biomedical Imaging, Department of Radiology and Neuroradiology, University Hospital Schleswig‐Holstein Christian‐Albrechts‐Universität zu Kiel Kiel Germany; ^3^ Siemens Healthineers AG Forchheim Germany

**Keywords:** brain clearance, diffusion MRI, DTI‐ALPS‐index, glymphatic system, image registration, Neurofluids

## Abstract

**Purpose:**

The aim of this work is to make the DTI along the perivascular space (DTI‐ALPS‐index) more robust with respect to region selection and the orientation of the head and fibers. We propose to address this matter by using the principal diffusion directions and an automated, atlas‐based region of interest (ROI) placement.

**Methods:**

Simulations were used to determine the dependence of the DTI‐ALPS‐index on the orientations of the head and nerve fibers. Human MRI was performed on 12 healthy volunteers at 3T using a 64 channel head coil (Cima.X Siemens), and the DTI‐ALPS‐index was calculated along the principal diffusion directions (ALPS‐PAS) and along the field of view or laboratory frame (ALPS‐LAB). To calculate the DTI‐ALPS‐index with reduced bias in native space, we developed a novel algorithm for an automatic ROI placement technique. Its calculated index results  were compared to those obtained from a manual ROIplacement in native space and from an existing atlas‐based ROI placement. Test–retest scans with varying head rotation were conducted for validation.

**Results:**

Simulations showed that ALPS‐PAS was more robust toward head and fiber rotations than ALPS‐LAB. In vivo, ALPS‐PAS yielded a 10% higher index than ALPS‐LAB. The automated ROI placement led to a smaller difference in the DTI‐ALPS‐index between test and retest measurement compared to the manual ROI placement. Stability was enhanced with ALPS‐PAS for all ROI placements and varying head rotation.

**Conclusion:**

Using the principal components of the diffusion and automated ROI selection increased the measured DTI‐ALPS‐index, improved the robustness toward head and fiber orientations and eliminated the need for manual region selection.

## 1 INTRODUCTION

The removal of metabolic waste and transport of solutes are vital for brain health.^1^ Evidence suggests that this process is facilitated by a glial lymphatic (glymphatic) system, though it remains difficult to identify both in vivo and through histological analysis. Waste from neuronal activity includes neurotransmitter byproducts, misfolded proteins, and cell debris.^1^, ^2^

The glymphatic system likely operates within the perivascular space (PVS), where aquaporin‐4 (AQP4) channels support water exchange with the brain parenchyma, clearing solutes and proteins such as amyloid‐beta.^3^

MRI based methods were suggested to investigate selected aspects of this system.^4^ These include methods with^5^ and without Gadolinium‐based contrast agents (GdCA). GdCA offers strong, lasting signals but may not reflect AQP4 activity.^1^ Methods without GdCA image water molecules, but may suffer from less signal and a shorter observation window for example arterial spin labeling (ASL),^6^ CEST,^7^ intravoxel incoherent motion (IVIM),^8^ and DTI along the PVS (DTI‐ALPS‐index).^9^, ^10^, ^11^

The DTI‐ALPS‐index is a measure for anisotropic water diffusion perpendicular to the main bundles of nerve fibers in selected regions of the brain. In selected regions, the diffusion weighted signal perpendicular to the main fibre orientation and along the postulated microvasculature was found to be 40% smaller than across the postulated microvasculature (in healthy controls^9^). The index was found to decrease with dementia and age,^9^ suggesting a less functional brain clearance.

Although these results may indicate a glymphatic flow along the perivascular spaces in selected areas of the human brain, recent studies suggest that the measured radial asymmetry of the diffusion tensor is largely influenced by axonal microstructure and crossing fibers, with only a minor contribution from perivascular spaces.^12^, ^13^, ^14^, ^15^ Still, the DTI‐ALPS‐index remains in use because it is acquired quickly and partially interpretable results, motivating efforts to improve its robustness.^13^

**FIGURE 1:**
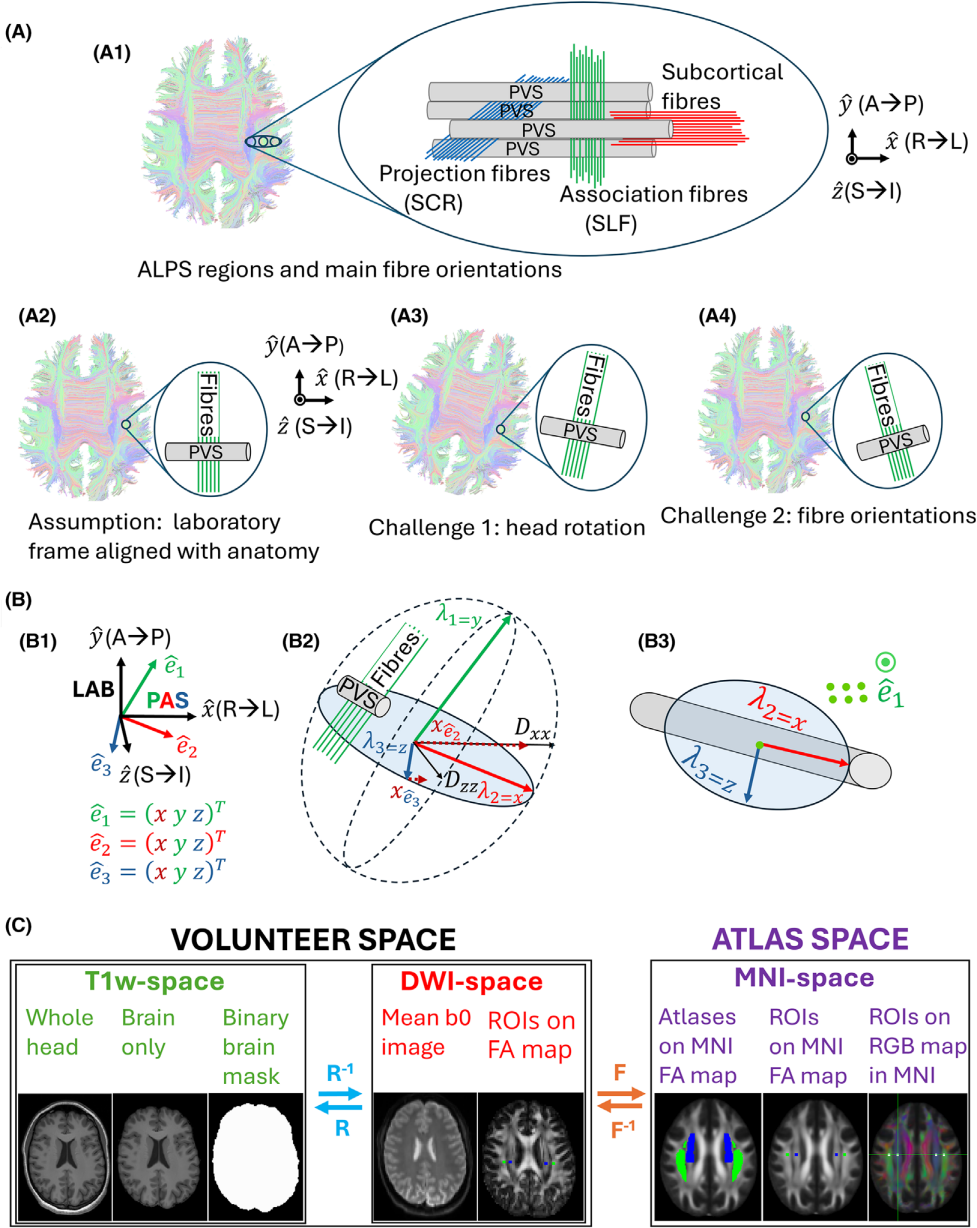
(A1) Left: RGB color‐coded tractography map (subject 1) with the colors belonging to the image's axes, also known as the laboratory frame (LAB) of reference ($\hat{x}$, $\hat{y}$, $\hat{z}$) is shown as coordinate system in black. Right: Idealized scheme of the vasculature with assumed PVS (gray) and dominant fibers in three areas used for ALPS (ALPS‐fiber‐regions): projection (blue), association (green) and subcortical (red). The colors from the original ALPS idea^9^ are used. (A2) In ALPS‐LAB, the directions of the fibers and PVS were assumed to coincide with the laboratory frame and therefore, the ALPS‐colors coincide with the colors of the tractography map. (A3) Challenge 1: Head rotation causes the ALPS‐fibers and the PVS to not align with the laboratory frame. (A4) Challenge 2: Even without head rotation, ALPS‐fibers and the PVS may not necessarily coincide with the laboratory frame. (B1) The PAS has the eigenvectors of the diffusion tensor ($\hat{e}$_1_, $\hat{e}$_2_, $\hat{e}$_3_) as its eigenbasis and is here represented in the colors of the ALPS‐fiber‐regions due to this work's assumption, that the ALPS‐fibers are more likely aligned with the PAS than the laboratory frame, eliminating the bias arising from challenges 1 (A3) and 2 (A4). (B2) An exemplary diffusion tensor with ${\hat{e}}_{1}$ pointing along the Association‐fibers. As ${\hat{e}}_{2}$ has a larger x‐component than ${\hat{e}}_{3}$ ($x_{{\hat{e}}_{2}}>x_{{\hat{e}}_{3}}$), the ALPS‐PAS equation (Eq. 4) uses $\lambda_{2}=\lambda_{x}$ and $\lambda_{3}=\lambda_{z}$. (B3) Bird's eye perspective from ${\hat{e}}_{1}$ to better visualize the orientation of the PVS in a plane perpendicular to ${\hat{e}}_{1}$. (C) Schematic view of the workflow used for the automatic evaluation of ALPS‐LAB and ALPS‐PAS in the volunteer DWI and atlas MNI spaces. In volunteer space, the matrix R/R^−1^ was used to transform the brain mask from T1w space to DWI space. The matrix F/F^−1^ was used to transform between the volunteer DWI and atlas MNI space. Four volumes of size 4 × 4 × 4 mm^3^ in the SCR and SLF were selected in the MNI space.

The original implementation of DTI‐ALPS‐index used the diffusion in the Cartesian directions of the laboratory frame (LAB), the Cartesian coordinates of the image, to determine the index. However, the LAB is not necessarily aligned with the nerve fibers and therefore the PVS in the brain; head and fiber orientation will affect the diffusion metrics^9^ and thus the DTI‐ALPS‐index (Figure 1A).

To correct head orientations, measured data was registered to a standard head orientation.^16^, ^17^ This registration requires heavy processing of the DTI data, which may affect the metrics, and does not account for individual fiber orientations (for example, the FMRIB58 template in Montreal Neurological Institute [MNI] space). A method to calculate the DTI‐ALPS‐index in the untransformed patient space, while correcting for the head orientation and taking the fiber orientation into account, however, was not reported.

Here, we propose to measure the anisotropy with respect to the main fiber orientation instead of the Cartesian coordinates of the image to enhance sensitivity to PVS orientation. We determined the main fiber orientation by calculating the main diffusion directions in a principal axis system (PAS)^12^, ^18^, ^19^ (Figure 1B) and tested the new method in a volunteer study.

Another key factor to determine the DTI‐ALPS‐index reliably is the (reproducible and precise) selection of the region of interest (ROI) where the index is calculated. The placement of the ROI varies with user input, scanner, and protocol.^13^, ^20^ However, transforming the acquired data to an atlas with preselected regions^17^ involves non‐rigid transformations with many degrees of freedom and strongly affects the measured MRI data.^21^

Here, we propose to calculate DTI‐ALPS‐index in regions defined on an atlas and transferred to the measured images in native space. This automatic ROI placement was compared to user‐selected ROI placement in native space as well as to ROI placement in atlas space.^16^, ^17^

We hypothesize that these methods (a) automatically measure the index with higher sensitivity to PVS orientation and (b) improve robustness in native space.

## 2 METHODS

### 2.1 DTI‐ALPS‐index computation

The diffusion tensor $\hat{D}$ in the laboratory frame of reference (LAB) can be described as

(1) $$\hat{D}=\left[\begin{array}{lll} D_{\mathrm{xx}} & D_{\mathrm{xy}} & D_{\mathrm{zz}}\\ D_{\mathrm{yx}} & D_{\mathrm{yy}} & D_{\mathrm{yz}}\\ D_{\mathrm{zx}} & D_{\mathrm{zy}} & D_{\mathrm{zz}} \end{array}\right],$$

where x, y, and z are the indices of the diffusion coefficients and refer to the Cartesian coordinates of the image. In this frame of reference (LAB), the DTI‐ALPS‐index (ALPS‐LAB) was introduced in Ref. 9 as:

(2) $$\text{ALPS}‐\mathrm{LAB}=\frac{\bar{D_{\mathrm{xx},\text{proj}},D_{\mathrm{xx},\text{assoc}}}}{\bar{D_{\mathrm{yy},\text{proj}},D_{\mathrm{zz},\text{assoc}}}},$$

where proj describes the superior corona radiata (SCR) region in the brain, in which projection fibers are dominant, and assoc describes the superior longitudinal fasciculus (SLF) region in the brain where association fibers are dominant (Figure 1A1). SCR and SLF refer to the ALPS‐fiber‐regions.

To reduce head and fiber orientation dependence and enhance PVS sensitivity (Figure 1A2–A4), we propose to compute the DTI‐ALPS‐index in the principal axis system (PAS). Diagonalizing $\hat{D}$ yields $\hat{\Lambda }$, which translates to a transformation of the coordinate system, resulting in the PAS or principal diffusion directions as the eigenbasis of the new coordinate system.^22^ $\hat{\Lambda }$ is defined as

(3) $$\hat{\Lambda }=\left[\begin{array}{lll} \lambda_{1} & 0 & 0\\ 0 & \lambda_{2} & 0\\ 0 & 0 & \lambda_{3} \end{array}\right],$$

where the eigenvalues of $\hat{D}$ are in the corresponding order: $\lambda_{1}\geq \lambda_{2}\geq \lambda_{3}$ (Figure 1B). The diagonalized matrix is used to calculate ALPS‐PAS^18^, ^19^:

(4) $$\text{ALPS}‐\mathrm{PAS}=\frac{\;\bar{\lambda_{x,\text{proj}},\lambda_{x,\text{assoc}}}\;}{\bar{\lambda_{y,\text{proj}},\lambda_{z,\text{assoc}}}},$$

where $\lambda_{x}$ is $\lambda_{2}$ or $\lambda_{3}$ depending on whose eigenvector has the larger x‐component, and $\lambda_{y}$ (projection) or $\lambda_{z}$ (association) is the remaining $\lambda_{2}$ or $\lambda_{3}$. This way, analogous to ALPS‐LAB (Eq. 2), the diffusion value along (potential) PVS is in the nominator, whereas the dominator includes the diffusion perpendicular to it (Figure 1B2, 1B3). However, Figure 1B1 illustrates that the ratio of the eigenvalues should differ from the ratio of the diffusion coefficients which should result in different ALPS‐LAB and ALPS‐PAS values. Note that $\lambda_{1}$ belongs to the main diffusion direction along the dominant fibers. Based on the original idea of DTI‐ALPS,^9^ the primary diffusion direction is not used to calculate the DTI‐ALPS‐index.

In the ALPS‐LAB and ALPS‐PAS calculations, the diffusion coefficients ($D_{\mathrm{xx}}$, $D_{\mathrm{yy}}\;\text{and}\;D_{\mathrm{zz}}$) or ($\lambda_{1},\lambda_{2}\;\text{and}\;\lambda_{3}$), were selected from the tensors in the voxels of the ROIs described later in this chapter. The overbars in Eq. (4) indicate the averages across the voxels in the ROIs. The use of eigenvalues in this calculation is consistent with previous work that considered diffusion asymmetries based on radial eigenvalues in ALPS‐fiber‐regions.^12^

### 2.2 Simulation experiments and Cardan‐angles

We performed simulations to investigate, how strongly ALPS‐LAB is affected by a mismatch between fiber and image orientation. We assumed a diffusion tensor $\hat{D}$ in the projection area, which is diagonal and perfectly aligned in the laboratory frame with its eigenvalues of 1.7, 0.4, and 0.2 μm^2^/ms. We applied selected rotations to the coordinate system of $\hat{D}$ (rotations of individual or all axes, defined by the Cardan‐angles (*Ψ* = (−180:180], *Θ* = [−90:90], *Φ* = (−180:180])^23^ for the intrinsic rotation (ZY′ X″), Figure 2.1, 2.3). We calculated ALPS‐PAS by dividing the second eigenvalue by the third and ALPS‐LAB by dividing $D_{\mathrm{xx}}$ by $D_{\mathrm{yy}}$, as the simulated initial tensor has an orientation like the tensors of the projection fibers.

**FIGURE 2:**
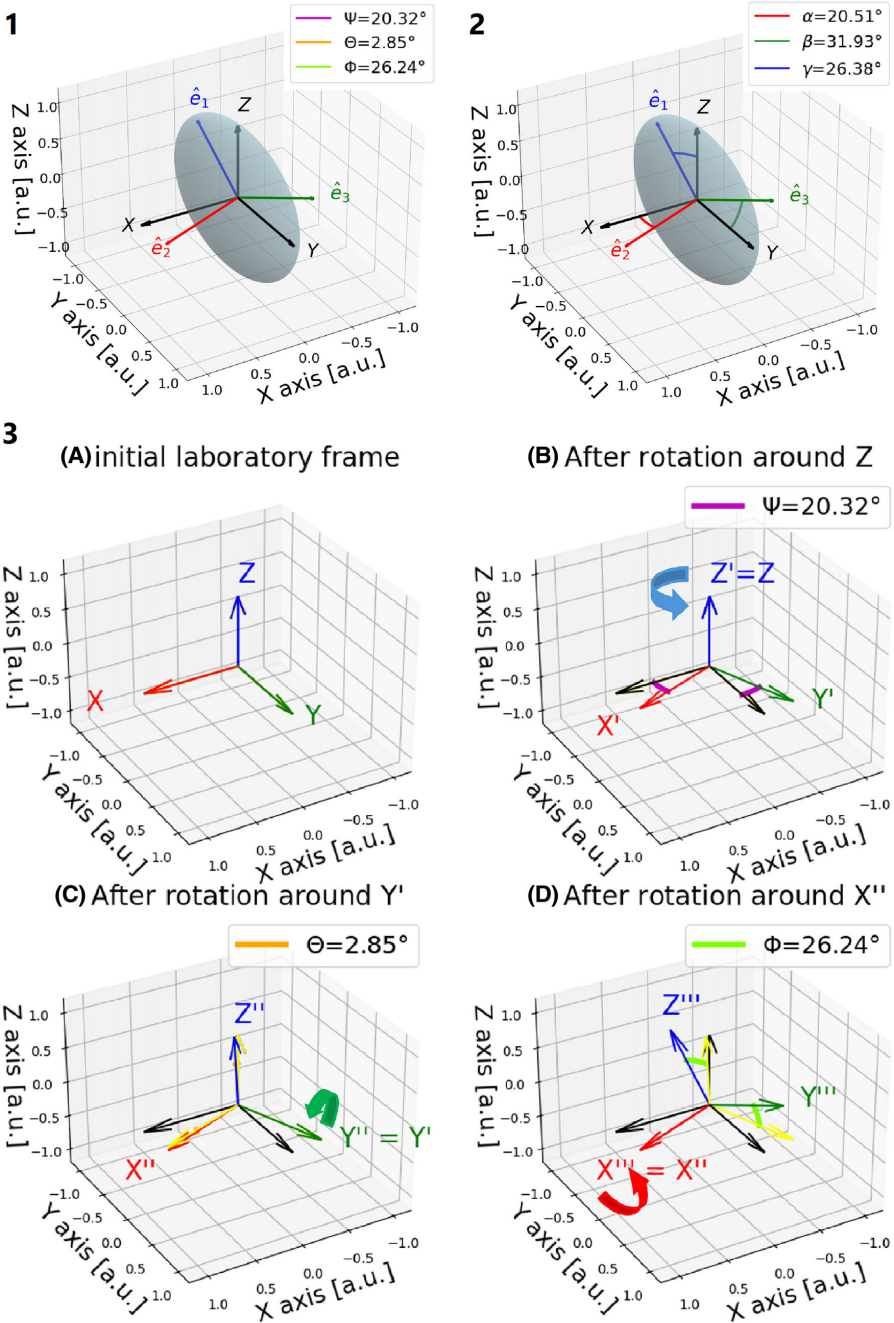
(1) Illustration of an exemplary diffusion tensor from a voxel in the SCR area of a volunteer with eigenvalues in [μm^2^/ms] $\lambda_{1}=1.06$, $\lambda_{2}=0.54$, $\lambda_{3}=0.35$ and eigenvectors ${\hat{e}}_{1}$ = (−0.93, −0.34, 0.04)^T^, ${\hat{e}}_{2}$= (0.29, −0.84, −0.44)^T^, ${\hat{e}}_{3}$= (−0.19, 0.39, −0.89)^T^. The transformation between the Cartesian laboratory axes and the eigenvectors (or principal axis) of the diffusion tensor can be described by three subsequent Cardan‐rotations Z, Y′, X″ (3A‐D). The minimum (absolute) angles between the PAS and laboratory frame are shown in 2.

Without rotation, ALPS‐LAB is ideal and identical to ALPS‐PAS. We used the ratio of ALPS‐LAB/ALPS‐PAS to assess the similarity of both indices.

### 2.3 In vivo experiments

In vivo measurements were acquired at 3 T (MAGNETOM Cima.X, Siemens Healthineers AG, Forchheim, Germany) with a maximum gradient amplitude of 200 mT/m, a maximum slew rate of 200 T/m/s, a two‐channel body transmit coil and 64‐channel receive head‐neck coil. Using a slice‐selective, diffusion weighted spin‐echo sequence with EPI readout, 66 diffusion volumes were acquired (b = [0,1000] s/mm^2^, diffusion gradient directions = [6, 30], averages = [1,2]) plus six *b* = 0 s/mm^2^ images with reversed phase encoding direction (main phase encoding direction: A➔P, without static field correction),  FOV = 220 × 220 mm^2^, 50 axial slices aligned to the anterior commissure‐posterior commissure (AC‐PC) line with 2 mm thickness, 2 mm isotropic resolution, matrix size 110 × 110 × 50, TE = 41 ms, volume TR = 6600 ms, readout bandwidth = 1698 Hz/Px, flip angle = 90°, GRAPPA factor 2, 28 reference lines, phase partial Fourier 0.75, effective echo spacing = 0.3 ms, no slice gap. In addition, a 3D T1‐weighted (T1w) MP‐RAGE image was acquired (FOV = 256 × 256 × 192 mm^3^, 1 mm isotropic resolution, TE = 2.45 ms, TR = 1900 ms, TI = 900 ms, flip angle = 9°, bandwidth = 170 Hz/Px). The entire imaging protocol required approximately 13 min.

The protocol was applied to 12 healthy subjects (age: 21–49, seven females and five males). The study was approved by the local ethics committee, and all participants gave written consent. Test–retest measurements were conducted on five subjects with a 5‐min break in between and five subjects were measured with and without deliberate head rotation to the top‐right. Subject 1 performed heavy head rotation.

### 2.4 Image processing

T1w images were corrected for bias fields, denoised and used to generate a brain mask (ANTs,^24^ Figure 1C). DWI post‐processing included denoising, unringing (Mrtrix^25^), distortion and eddy current corrections (FSL^21^). A transformation matrix R between T1w and DWI images was calculated and used to transform the brain mask to the DWI data (Epireg^21^). The diffusion tensor and color‐coded FA (RGB) maps (Dipy^26^) were calculated on the masked diffusion data. The brain‐masked DWI data was transformed to the MNI atlas space by calculating a transformation matrix F using the FA maps of both spaces with linear and non‐linear transformations (flirt, fnirt).^21^ The transformation matrix F was applied onto the DTI metrics (flirt, fnirt, Vecreg).^21^

### 2.5 ROIs

We calculated ALPS‐LAB and ALPS‐PAS for the left and the right hemisphere by creating ROIs (volume: 4 × 4 × 4 mm^3^) in the ALPS‐fiber‐regions in three different ways. For each way, four ROIs were defined: one in the SCR and one in the SLF, on both the left and right sides (Figure 1C).

- MNI ROIs (existing atlas based automated way^17^): The four ROIs were placed on a brain template (FMRIB58_FA_1mm, MNI152 space^27^, ^28^, ^29^).
- Manual ROIs in DWI space (the way DTI‐ALPS‐index was introduced^9^): An experienced radiologist selected the ROIs manually on the volunteer RGB maps.
- MNI ROIs in DWI space (novel technique of this work): the MNI‐ROIs from (A) were transformed to the DWI space using F^−1^ by a custom algorithm, which was applied to retain the structural integrity of the ROIs. This algorithm ensures that the ROIs remain square, with a voxel size of 2 × 2 × 2 voxels (corresponding to 4 × 4 × 4 mm^3^). If transformation results in more than two voxels in a given direction, the two voxels with the highest intensity were retained. If only one voxel remains, the missing voxel was determined by selecting the neighboring voxel with the highest FA value.

In the following, we refer to the “MNI ROIs”, “Manual ROIs in DWI space”, and “MNI ROIs in DWI space” as “ROI‐options”.

For each subject and in each of the four ALPS‐fiber‐regions, the individual distance between the centers of mass (*Δ*) for ROI‐options B and C in DWI space was calculated, followed by the mean and SD across subjects.

### 2.6 Angles and ALPS‐LAB, ALPS‐PAS

The orientation of the diffusion tensor was described using intrinsic Cardan‐angle rotations for each voxel within the ROI, as well as by the mean angles across all voxels within the ROI (ZY′ X″ with the angles *Ψ*, *Θ*, *Φ*, Figure 2.1, 2.3).

Similarly, the absolute angles between the laboratory axes and the principal axes of the diffusion tensors were calculated (Figure 2.2).

ALPS‐LAB and ALPS‐PAS were calculated for each subject and each hemisphere (Eqs. 2 and 4), for all ROI‐options.

### 2.7 Statistical evaluation

The mean ALPS‐LAB/ ALPS‐PAS ratio and relative SD (coefficient of variance, CV) was computed for all subjects and all ROI‐options (A, B, C).

Two‐tailed, paired *t*‐tests with significance level *p* = 0.05 enabled a comparison of ALPS‐LAB and ALPS‐PAS for each ROI‐option independently. To increase test validity, indices of both brain hemispheres were combined. Separate *t*‐tests were conducted to compare the ALPS‐PAS values across the different ROI‐options; likewise, the ALPS‐LAB values were compared separately.

For retest and rotation measurements the ratio ρ = new index/ old index together with mean and SD of ρ across subjects were calculated.

## 3 RESULTS

**FIGURE 3:**
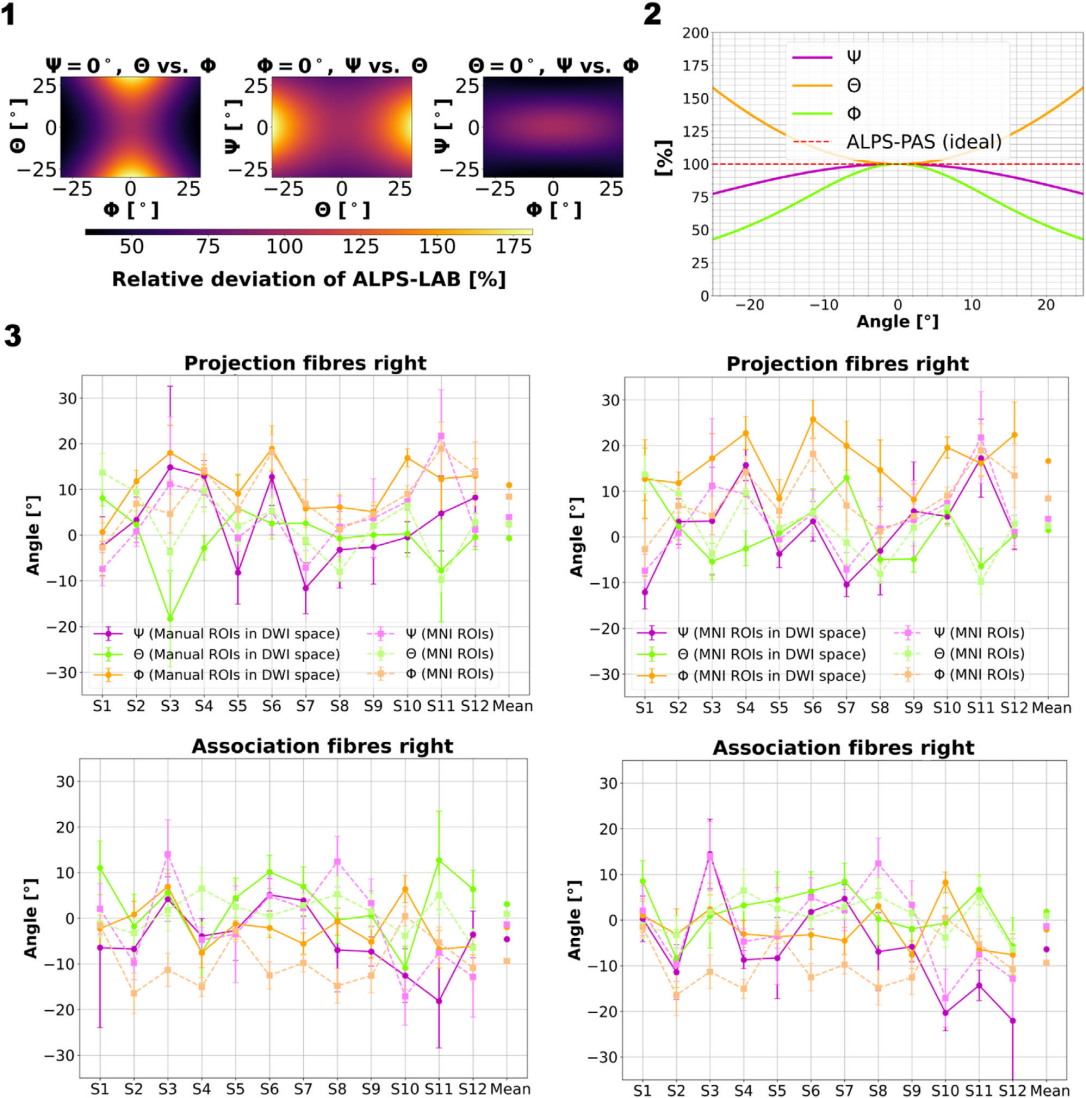
Simulated effect of fiber orientations on the ALPS‐index (1, 2) and measured fiber orientations in the volunteers (3). (1) Effect of rotations on the DTI‐ALPS‐index. Three simulated Cardan‐angle combinations; in each subfigure one angle is set to zero, whereas the other two are simulated for a selected Cardan‐angle range. (2) Similar to (1) but setting two Cardan‐angles to zero while varying the third in the same angle range. (3) The curves represent the mean and SD values of the three Cardan‐angles computed for each voxel within the ROIs, which are defined per ALPS‐fiber‐region, for all subjects. For each angle, the mean and SD were calculated across voxels within each ROI for each subject. Subsequently, these values were averaged across subjects. The results shown are for the right hemisphere as an illustration. The colors violet, chartreuse, and orange characterize (ψ,θ,ϕ) in DWI space (Left: Manual ROIs in DWI space; Right: MNI ROIs in DWI space) and light violet, light chartreuse, and light orange characterize (ψ,θ,ϕ) in MNI space (MNI ROIs).

The DTI‐ALPS‐index was found to depend on the relative orientation of both systems (Figure 3).

For example, when all three angles were varied by ±5° to ±10°, ALPS‐LAB differed by at least 5% and up to 20% from ALPS‐PAS (Figure 3.1). A single *Ψ* = ±20° rotation around the Z‐axis caused the DTI‐ALPS‐index to decrease by 20% (Figure 3.2). The strongest effect was found for the rotation around the novel X″‐axis, where *Φ* = ±20° rotation resulted in 45% decreased DTI‐ALPS‐index.

The ratio of ALPS‐LAB/ALPS‐PAS simulated for the total range of Cardan‐angles is provided in Figure S1A,B.

In vivo, maximum Cardan‐angles of ±35° for all ROI‐options were observed for individual voxels, and ±20° for the ROI average (Figure 3.3). These angles were similar as the absolute angles between the Cartesian axis and the new principal axis (Figure S2). The magnitude of the deviations suggests that this effect can change the measured in vivo DTI‐ALPS‐index up to 30%.

Next, ALPS‐PAS and ALPS‐LAB were calculated for all ROI‐options (A, B, C) on the in vivo data and analyzed (Table 1). For the convenience of the reader, we labeled the individual data with descriptive names and indices, for example “ALPS‐LAB in MNI ROIs, left and right” as No. 1, “ALPS‐PAS in MNI ROIs in DWI space, left and right” as No. 6. We used these indices for labeling statistical metrics, for example “(two‐tailed, paired)” *t*‐test between (ALPS‐LAB in MNI ROIs left and right) and (ALPS‐PAS in MNI ROIs left and right) as “*t*‐test (1 vs. 2) = 1.15 · 10^−9^” or the “mean (ALPS‐LAB in MNI ROIs left and right)” as mean (1) = 1.53.

**TABLE 1:** For both, DWI (Manual ROIs in DWI (volunteer) space, MNI ROIs in DWI (volunteer) space) and MNI (atlas) space, the DTI‐ALPS‐index is calculated in the laboratory frame (ALPS‐LAB) and in the principal axis system (ALPS‐PAS) for each brain side (Left‐L, Right‐R).

	MNI ROIs (A)				Manual ROIs in DWI (volunteer) space (B)				MNI ROIs in DWI (volunteer) space (C)			
	No. 1		No. 2		No. 3		No. 4		No. 5		No. 6	
	ALPS‐LAB		ALPS‐PAS		ALPS‐LAB		ALPS‐PAS		ALPS‐LAB		ALPS‐PAS	
(a) TEST												
	**L**	**R**	**L**	**R**	**L**	**R**	**L**	**R**	**L**	**R**	**L**	**R**
S1 (f, 24)	1.60	1.61	1.82	1.72	1.71	1.68	1.93	1.72	1.70	1.54	1.94	1.71
S2 (m, 29)	1.49	1.55	1.64	1.65	1.72	1.60	1.88	1.70	1.50	1.64	1.62	1.75
S3 (m, 27)	1.56	1.36	1.72	1.43	1.63	1.28	1.76	1.35	1.64	1.41	1.79	1.46
S4 (f, 30)	1.39	1.34	1.65	1.52	1.46	1.51	1.66	1.74	1.37	1.37	1.64	1.60
S5 (f, 22)	1.81	1.78	1.91	1.84	1.78	1.77	1.90	1.74	1.83	1.83	1.93	1.88
S6 (m, 21)	1.40	1.46	1.71	1.77	1.43	1.37	1.70	1.51	1.50	1.43	1.84	1.72
S7 (m, 21)	1.68	1.59	1.97	1.79	1.35	1.57	1.67	1.67	1.79	1.59	2.03	1.87
S8 (f, 23)	1.62	1.55	1.85	1.63	1.69	1.62	1.83	1.63	1.69	1.59	1.92	1.68
S9 (m, 27)	1.51	1.41	1.72	1.48	1.60	1.56	1.78	1.60	1.67	1.35	1.86	1.40
S10 (f, 49)	1.55	1.71	1.97	1.85	1.71	1.71	2.14	1.90	1.51	1.73	1.96	1.90
S11 (f, 26)	1.39	1.33	1.74	1.53	1.53	1.35	1.73	1.41	1.42	1.43	1.71	1.60
S12 (f, 26)	1.50	1.56	1.68	1.75	1.42	1.54	1.63	1.67	1.52	1.57	1.72	1.80
Mean	**1.54**	**1.52**	**1.78**	**1.66**	**1.59**	**1.55**	**1.80**	**1.64**	**1.60**	**1.54**	**1.83**	**1.70**
CV [%]	**7.78**	**9.10**	**6.39**	**8.36**	**8.65**	**9.25**	**7.72**	**8.90**	**8.74**	**9.23**	**6.99**	**9.03**
Mean ± CV	**1.53 ± 0.13**		**1.72 ± 0.14**		**1.57 ± 0.14**		**1.72 ± 0.17**		**1.57 ± 0.15**		**1.76 ± 0.16**	
*t*‐tests	1.15 · 10^−9^ (1 vs. 2)*				2.04 · 10^−7^ (3 vs. 4)*				1.28 · 10^−9^ (5 vs. 6)*		
(*p* = 0.05)	0.15 (1 vs. 3)		0.98 (2 vs. 4)		0.97 (3 vs. 5)		0.13 (4 vs. 6)		0.01 (1 vs. 5)*		0.13 (2 vs. 6)	
(b) RETEST												
S2 index	**1.51**	**1.52**	**1.62**	**1.63**	**1.55**	**1.54**	**1.71**	**1.65**	**1.56**	**1.55**	**1.65**	**1.68**
ρ	**101.3**	**98.1**	**98.8**	**98.8**	**90.1**	**96.3**	**91.0**	**97.1**	**104.0**	**94.5**	**101.9**	**96.0**
S3 index	**1.56**	**1.36**	**1.73**	**1.44**	**1.59**	**1.49**	**1.73**	**1.59**	**1.64**	**1.37**	**1.80**	**1.46**
ρ	**100.0**	**100.0**	**100.6**	**100.7**	**97.5**	**116.4**	**98.3**	**117.8**	**100**	**97.2**	**100.6**	**100.0**
S4 index	**1.40**	**1.30**	**1.65**	**1.51**	**1.61**	**1.80**	**1.78**	**1.96**	**1.42**	**1.32**	**1.64**	**1.49**
ρ	**100.7**	**97.0**	**100.0**	**99.3**	**110.3**	**119.2**	**107.2**	**112.6**	**103.6**	**96.4**	**100.0**	**93.1**
S5 index	**1.82**	**1.82**	**1.88**	**1.87**	**1.85**	**1.68**	**1.93**	**1.76**	**1.81**	**1.83**	**1.91**	**1.89**
ρ	**100.6**	**102.2**	**98.4**	**101.6**	**103.9**	**94.9**	**101.6**	**101.1**	**98.9**	**100.0**	**99.0**	**100.5**
S6 index	**1.38**	**1.42**	**1.74**	**1.74**	**1.46**	**1.40**	**1.81**	**1.62**	**1.47**	**1.37**	**1.82**	**1.70**
ρ	**98.6**	**97.3**	**101.8**	**98.3**	**102.1**	**102.2**	**106.5**	**107.3**	**98.0**	**95.8**	**98.9**	**98.8**
Mean of ρ	**100.2**	**98.9**	**99.9**	**99.8**	**100.8**	**105.8**	**100.9**	**107.2**	**100.9**	**96.8**	**100.1**	**97.7**
SD of ρ	**0.94**	**1.97**	**1.21**	**1.23**	**6.72**	**10.15**	**5.95**	**7.50**	**2.46**	**1.83**	**1.09**	**2.77**
(c) ROTATED												
S1 index	**1.40**	**1.53**	**1.83**	**1.71**	**1.32**	**1.82**	**1.52**	**1.69**	**1.34**	**1.44**	**1.71**	**1.65**
ρ	**87.5**	**95.0**	**100.5**	**99.4**	**77.2**	**108.3**	**78.8**	**98.3**	**78.8**	**93.5**	**88.1**	**96.5**
S2 index	**1.53**	**1.54**	**1.62**	**1.62**	**1.52**	**1.65**	**1.58**	**1.71**	**1.59**	**1.60**	**1.67**	**1.67**
ρ	**102.7**	**99.4**	**98.8**	**98.2**	**88.4**	**103.1**	**84.0**	**100.6**	**106.0**	**97.6**	**103.1**	**95.4**
S5 index	**1.76**	**1.77**	**1.86**	**1.83**	**1.66**	**1.52**	**1.76**	**1.65**	**1.76**	**1.74**	**1.84**	**1.79**
ρ	**97.2**	**99.4**	**97.4**	**99.5**	**93.3**	**85.9**	**92.6**	**94.8**	**96.2**	**95.1**	**95.3**	**95.2**
S6 index	**1.46**	**1.44**	**1.75**	**1.76**	**1.40**	**1.40**	**1.66**	**1.63**	**1.59**	**1.41**	**1.90**	**1.70**
ρ	**104.3**	**98.6**	**102.3**	**99.4**	**97.9**	**102.2**	**97.6**	**107.9**	**106.0**	**98.6**	**103.3**	**98.8**
S7 index	**1.69**	**1.63**	**2.05**	**1.79**	**1.67**	**1.42**	**1.83**	**1.89**	**1.62**	**1.63**	**2.10**	**1.87**
ρ	**100.6**	**102.5**	**104.1**	**100.0**	**123.7**	**90.4**	**109.6**	**113.2**	**90.5**	**102.5**	**103.4**	**100.0**
Mean of ρ	**99.9**	**101.7**	**100.5**	**101.0**	**99.2**	**99.2**	**94.8**	**102.5**	**97.5**	**101.3**	**98.4**	**99.0**
SD of ρ	**7.5**	**6.9**	**2.5**	**2.8**	**15.1**	**9.5**	**9.9**	**6.9**	**12.8**	**8.3**	**5.9**	**3.2**

*Note*: Subject information is given in the first column including the gender (female‐f, male‐m) and age in years. The mean of each brain hemisphere, the coefficient of variance (CV) across subjects and the mean of combined brain hemispheres are included. Results of two‐tailed paired *t*‐tests between ALPS‐PAS and ALPS‐LAB values, combining both brain hemispheres, are provided for each individual ROI‐option in an additional row (significance is marked with an asterisk). Furthermore, *t*‐tests were also performed to compare ALPS‐PAS values between different ROI‐options, and analogously for ALPS‐LAB values. Note that the combinations of ROI selection and index calculation methods are labeled No. 1–6 and are used to identify the statistical comparisons (e.g., p(1 vs. 2)). For robustness, the indices of retest scans of five selected subjects are added to the table including the ratio ρ = retest/test in percent. The distribution of ρ is described by the mean and SD, both in percent as well. The same is presented for retest scans with varying head rotation. Bold values denote means and SDs for readability.

Comparing ALPS‐PAS and ALPS‐LAB, we found that the mean ALPS‐PAS was higher and statistically different than ALPS‐LAB for all ROI‐options, on average by 14% in the left hemisphere and 8% in the right hemisphere, with *p* < 2· 10^−7^ (Table 1). The CV was similar for ALPS‐LAB and ALPS‐PAS, with a tendency for less variance in the laboratory frame.

Comparing the different ROI‐options, we found that the ROI‐options B and C were placed in the ALPS‐fiber‐regions (SCR and SLF) for all measurements with normal head positioning (an example is presented in Figure S3). The distance of the centers of mass between the ROI‐options B and C was (3.3 ± 2 mm) on average, with extremes of 0 and 10.4 mm (Table 2). The ALPS‐indices across the ROI‐options (within one reference frame) were not statistically different except for ALPS‐LAB, calculated in ROI‐option A and ROI‐option C (p(1 vs. 5) = 0.01 in Table 1).

**TABLE 2:** Calculated distance between the center of mass for ROI‐option B and ROI‐option C in each ALPS‐fiber‐region (*Δ*1–*Δ*4) for all subjects.

	Δ1 [mm] (R‐Assoc)	Δ2 [mm] (R‐Proj)	Δ3 [mm] (L‐Assoc)	Δ4 [mm] (L‐Proj)
TEST				
S1	2.83	2.83	2.83	2.00
S2	2.00	0.00	2.83	2.83
S3	2.00	2.83	2.00	2.83
S4	4.00	4.90	2.00	4.90
S5	4.47	2.00	0.00	2.83
S6	2.00	2.00	4.47	4.00
S7	2.00	2.83	4.47	4.47
S8	2.00	4.47	2.00	4.47
S9	2.00	2.00	3.46	0.00
S10	4.00	6.00	2.00	2.00
S11	10.39	10.20	6.32	8.25
S12	4.47	2.00	2.83	2.00
Mean	**3.51**	**3.51**	**2.93**	**3.38**
SD	**2.31**	**2.53**	**1.54**	**1.98**
RETEST				
S2	2.83 (+0.83)	2.00 (+2.00)	2.00 (−0.83)	3.46 (+0.63)
S3	2.00 (±0.00)	2.00 (−0.83)	2.83 (+0.83)	2.83 (±0.00)
S4	8.25 (+4.25)	8.94 (+4.04)	4.00 (+2.00)	6.32 (+1.42)
S5	4.47 (±0.00)	4.90 (+2.90)	2.83 (+2.83)	0.00 (−2.83)
S6	0.00 (−2.00)	2.00 (±0.00)	0.00 (−4.47)	0.00 (−4.00)
Mean	**3.51 (+0.62)**	**3.97 (+1.62)**	**2.33 (+0.07)**	**2.52 (−0.96)**
SD	**2.77 (+1.67)**	**2.73 (+1.15)**	**1.33 (−0.12)**	**2.37 (+1.53)**
ROTATED				
S1	6.63 (+3.80)	6.32 (+3.49)	2.83 (±0.00)	4.47 (+2.47)
S2	2.00 (±0.00)	2.00 (+2.00)	2.83 (±0.00)	2.00 (−0.83)
S5	4.90 (+0.43)	2.83 (+0.83)	2.00 (+2.00)	2.83 (±0.00)
S6	2.00 (±0.00)	4.90 (+2.90)	2.83 (−1.64)	2.00 (−2.00)
S7	8.25 (+6.25)	8.25 (+5.42)	2.00 (−2.47)	2.83 (−1.64)
Mean	**4.76 (+1.70)**	**4.86 (+1.95)**	**2.50 (−0.25)**	**2.83 (−0.81)**
SD	**2.49 (+1.47)**	**2.28 (+1.22)**	**0.41 (−1.27)**	**0.90 (−0.17)**

*Note*: On average, the distances were about (3.4 ± 2.1) mm, and the DTI‐ALPS‐indices in these regions were not statistically different (Table 1). In addition, *Δ*1–*Δ*4 of retest scans with and without varying head rotation are placed below with the difference in [mm] to the test measurements in brackets. The means and SDs of *Δ*1–*Δ*4 and their differences are demonstrated. Bold values denote means and SDs for readability.

For test ‐retest scans (Table 1), the mean of ρ was close to 100% for ROI‐options A and C with a SD below 2% whereas for ROI‐option B the mean of ρ deviated more strongly from 100%. Similar results were obtained for rotation scans with increased SD. Notably, ROI‐option A did not result in ρ = 100% for ALPS‐LAB. In native space, using ALPS‐PAS reduced the deviation strongly.

Transferring the predefined ROIs from MNI to the DWI space (ROI‐option C) placed the ROIs within the ALPS‐fiber‐regions, with and without head rotation (Figure S4). At the same time, transferring the image data to the MNI space resulted in significant changes to the anatomy (Figure S4).

*Δ*1‐*Δ*4 of the retest‐ and rotation scans did not differ more than 2 mm (voxel size) from the *Δ*1–*Δ*4 of the test scans (Table 2).

## 4 DISCUSSION

We introduced novel methods to make the DTI‐ALPS‐index less susceptible to individual head and fiber orientations, and less susceptible for individual ROI selection. We will discuss both methods in the following.

As expected, simulations confirmed that the DTI‐ALPS‐index is sensitive to the alignment between brain anatomy and evaluation axes: even small angular deviations, which are common in vivo, can substantially affect the index. Whether or not it increases the value of the DTI‐ALPS‐index depends on the orientation with respect to the main diffusion direction. As we simulated a diffusion tensor in the projection area, the first eigenvector points along the positive Z‐axis and the second/third eigenvector points along the positive X‐axis/ Y‐axis. A rotation around the Y‐axis with *Θ* = 90°, for example, rotates the first eigenvector ${\hat{e}}_{1}$ to the positive X‐axis and the second eigenvector ${\hat{e}}_{2}$ to the negative Z‐axis. The third eigenvector ${\hat{e}}_{3}$ still points along the positive Y‐axis. Therefore, for this rotation, ALPS‐LAB = $D_{\mathrm{xx}}$/$D_{\mathrm{yy}}$ = $\lambda_{1}$/$\lambda_{3}$ > ALPS‐PAS = $\lambda_{2}$/$\lambda_{3}$.

In vivo, we found that deviations around 10° are not uncommon between anatomy (measured by diffusion) and LAB, thus the x‐component of ${\hat{e}}_{2}$ was always larger than the one of ${\hat{e}}_{3}$ ($\lambda_{x}=\lambda_{2}$ in Eq. 4), which is in agreement with Ref. 12 and still requires elaboration.

The higher ALPS‐PAS values compared to ALPS‐LAB across all ROI‐options suggests higher sensitivity of the DTI‐ALPS‐index to PVS orientation. In addition, test‐retest measurements, also with heavy head rotation, support this finding and showed higher reproducibility for ALPS‐PAS than ALPS‐LAB. Despite confounding effects from tissue microstructure and fiber crossings (Refs. 12, 13), PAS‐based evaluation appears more reliable and effective than LAB. However, recent studies show that advanced modeling approaches can mitigate, but not fully eliminate, such effects, which therefore remain a methodological limitation.^14^, ^15^ Validation in larger cohorts, together with the use of these reported methods, would further strengthen the PAS‐based calculation.

Regarding the selection of ROIs for DTI‐ALPS‐index calculation, all ROI‐options yielded index values expected for healthy controls with no statistical differences, except for the *t*‐test (1 vs. 5). However, ROI‐options A and C were determined fully automatically in contrast to the manual placement used in ROI‐option B, which is operator‐dependent. Notice that ROI‐options A and C require image registration. Whereas in ROI‐option C, the registration effects only the positions of the binary ROIs, it involves heavy image processing for ROI‐option A. Since the ROIs are relatively small, this could strongly affect the DTI‐ALPS‐index. In this study, reproducibility was only assessed by comparing manual and automated ROIs; however, formal intra‐ and inter‐rater variability was not evaluated, which represents a limitation of the current work.

Retest measurements showed higher DTI‐ALPS‐index reproducibility for ROI‐options A and C, especially for ALPS‐PAS. The differences between ALPS‐LAB of test‐ and retest measurements for ROI‐option A indicated the negative effect of the erroneous smoothing effects, mentioned above. The strong change of ALPS‐LAB was particularly the case for heavy head rotation, performed by subject 1, as here the registration to the atlas included stronger non‐rigid transformations on the diffusion data.

Continuing the analysis of ROI placement, the mean distance between the manually and automatically placed ROIs was about 3 mm, close to the voxel size. In contrast, retest measurements, conducted with and without head rotation, showed varying distances between the ROIs. The observed discrepancies likely stemmed from inconsistencies in manual ROI placement, as these led to greater deviations in DTI‐ALPS‐indices compared to automatically placed ROIs.

## 5 CONCLUSIONS

Using the principal diffusion directions (PAS) instead of the image directions (LAB) to calculate the DTI‐ALPS‐index increased its value by up to 20%, improved the test–retest results and compensated for head mispositioning. Automated ROI placement did not significantly change the indices, but was more robust than manual placement and did not require manipulating the image data.

## CONFLICT OF INTEREST STATEMENT

The author Ali Ajouz declares no potential conflict of interests. Ali Ajouz is an employee of Siemens Healthineers AG and UKSH. The author Lynn Johann Frohwein declares no potential conflict of interests. Lynn Johann Frohwein is an employee of Siemens Healthineers AG. The author Naomi Larsen declares no potential conflict of interests. The author Olav Jansen declares no potential conflict of interests. Olav Jansen is an employee of UKSH. The author Svea Seehafer declares no potential conflict of interests.

## Supporting information


**Figure S1.** (A) In addition to Figure 3.1, 3.2, the relation of ALPS‐LAB to ALPS‐PAS for multiple Cardan‐angle combinations with a simultaneous change of all angles is plotted. (B) Similar to (A) but setting two Cardan‐angles to zero while varying the third in the entire Cardan‐angle range.
**Figure S2.** The curves represent the mean and standard deviation values of the three absolute angles computed for each voxel within the ROIs, which are defined per ALPS‐fiber‐region. For each angle, the mean and standard deviation were calculated across voxels within each ROI for each subject. Subsequently, these values were averaged across subjects. The results shown are for the right hemisphere as an illustration. The colors blue, green and red characterize (α,β,γ) in DWI space (Left: Manual ROIs in DWI space; Right: MNI ROIs in DWI space) and light blue, light green and light purple characterize (α,β,γ) in MNI space (MNI ROIs).
**Figure S3.** A 3D RGB‐encoded map of diffusion images with manually (red, ROI‐option B) and automatically (white, ROI‐option C) placed ROIs in the native imaging space (DWI space) for subject one.
**Figure S4.** Exemplary RGB maps in MNI space (upper) with MNI ROIs (white squares ROI‐option A) and in DWI space (down) with Manual ROIs in DWI space (red squares ROI‐option B) and MNI ROIs in DWI space (white squares ROI‐option C) of subject one, with (right) and without (left) head rotation. In all cases, the algorithm placed the ROIs (ROI‐option C) in anatomically meaningful areas (ALPS‐fiber‐regions green and blue areas). Note the smoothing effect of transforming the data to the MNI space.

## Data Availability

Research data are not shared.

## References

[1] Iliff JJ , Wang M , Liao Y , et al. A Paravascular pathway facilitates CSF flow through the brain parenchyma and the clearance of interstitial solutes, including amyloid *β* . Sci Transl Med. 2012;4:147ra111. doi:10.1126/scitranslmed.3003748 PMC3551275 22896675

[2] Bohr T , Hjorth PG , Holst SC , et al. The glymphatic system: current understanding and modeling. iScience. 2022;25:104987. doi:10.1016/j.isci.2022.104987 36093063 PMC9460186

[3] Smith AJ , Verkman AS . The “glymphatic” mechanism for solute clearance in Alzheimer's disease: game changer or unproven speculation? FASEB J. 2018;32:543‐551. doi:10.1096/fj.201700999 29101220 PMC5888402

[4] Kamagata K , Saito Y , Andica C , et al. Noninvasive magnetic resonance imaging measures of Glymphatic system activity. Magn Reson Imaging. 2024;59:1476‐1493. doi:10.1002/jmri.28977 37655849

[5] Taoka T , Naganawa S . Glymphatic imaging using MRI. Magn Reson Imaging. 2020;51:11‐24. doi:10.1002/jmri.26892 31423710

[6] Joseph CR , Benhatzel CM , Stern LJ , Hopper OM , Lockwood MD . Pilot study utilizing MRI 3D TGSE PASL (arterial spin labeling) differentiating clearance rates of labeled protons in the CNS of patients with early Alzheimer disease from normal subjects. Magn Reson Mater Phy. 2020;33:559‐568. doi:10.1007/s10334-019-00818-3 31897905

[7] Chen Y , Dai Z , Fan R , et al. Glymphatic system visualized by chemical‐exchange‐saturation‐transfer magnetic resonance imaging. ACS Chem Neurosci. 2020;11:1978‐1984. doi:10.1021/acschemneuro.0c00222 32492333

[8] Gomolka RS , Hablitz LM , Mestre H , et al. Loss of aquaporin‐4 results in glymphatic system dysfunction via brain‐wide interstitial fluid stagnation. elife. 2023;12:e82232. doi:10.7554/eLife.82232 36757363 PMC9995113

[9] Taoka T , Masutani Y , Kawai H , et al. Evaluation of glymphatic system activity with the diffusion MR technique: diffusion tensor image analysis along the perivascular space (DTI‐ALPS) in Alzheimer's disease cases. Jpn J Radiol. 2017;35:172‐178. doi:10.1007/s11604-017-0617-z 28197821

[10] Taoka T , Ito R , Nakamichi R , et al. Reproducibility of diffusion tensor image analysis along the perivascular space (DTI‐ALPS) for evaluating interstitial fluid diffusivity and glymphatic function: CHanges in Alps index on multiple conditiON acquIsition eXperiment (CHAMONIX) study. Jpn J Radiol. 2022;40:147‐158. doi:10.1007/s11604-021-01187-5 34390452 PMC8803717

[11] Taoka T , Ito R , Nakamichi R , Nakane T , Kawai H , Naganawa S . Diffusion tensor image analysis ALong the perivascular space (DTI‐ALPS): revisiting the meaning and significance of the method. Magn Reson Med Sci. 2024;23:268‐290. doi:10.2463/mrms.rev.2023-0175 38569866 PMC11234944

[12] Wright AM , Wu Y , Chen N , Wen Q . Exploring radial asymmetry in MR diffusion tensor imaging and its impact on the interpretation of Glymphatic mechanisms. Magn Reson Imaging. 2024;60:1432‐1441. doi:10.1002/jmri.29203 PMC11213825 38156600

[13] Haller S , Moy L , Anzai Y . Evaluation of diffusion tensor imaging analysis along the perivascular space as a marker of the Glymphatic system. Radiology. 2024;310:e232899. doi:10.1148/radiol.232899 38289215

[14] Taoka T , Iwamoto K , Miyata S , et al. Contribution of white matter microstructure to diffusion tensor image analysis along perivascular space in obstructive sleep apnea. Jpn J Radiol. 2025. doi:10.1007/s11604-025-01838-x PMC12647286 40705166

[15] Schilling KG , Newton A , Tax C , et al. White matter geometry confounds diffusion tensor imaging along perivascular space (DTI‐ALPS) measures. Hum Brain Mapp. 2025;46:e70282. doi:10.1002/hbm.70282 40622117 PMC12231058

[16] Tatekawa H , Matsushita S , Ueda D , et al. Improved reproducibility of diffusion tensor image analysis along the perivascular space (DTI‐ALPS) index: an analysis of reorientation technique of the OASIS‐3 dataset. Jpn J Radiol. 2023;41:393‐400. doi:10.1007/s11604-022-01370-2 36472803 PMC10066136

[17] Saito Y , Kamagata K , Andica C , et al. Reproducibility of automated calculation technique for diffusion tensor image analysis along the perivascular space. Jpn J Radiol. 2023;41:947‐954. doi:10.1007/s11604-023-01415-0 37162692

[18] Ajouz A . Impact of image registration and fibre orientation on the DTI‐ALPS‐index. Paper presented at: 26th Annual Meeting of the German Chapter of the International Society for Magnetic Resonance in Medicine 2024, Tübingen Tübingen, Germany; 2024:129–130.

[19] Ajouz A , Frohwein LJ , Jansen O , Hövener J‐B . Using a principle axis system allows automatic evaluation of glymphatic flow with ALPS in imaging space and corrects for head and fibre orientation. *Proceedings of the 33rd Annual Meeting of ISMRM & ISMRT*. ISMRM, Honolulu, Hawai‘i, USA; 2025:5279.

[20] Ulloa P , Rudolf JC , Kremer J , Schmidt A , Schramm P . Influence of orientation, size and shape of the region of interest in diffusion MRI along perivascular spaces index. Magn Reson Mater Phy. 2025. doi:10.1007/s10334-025-01248-0 PMC12497668 40214874

[21] Jenkinson M , Beckmann CF , Behrens TEJ , Woolrich MW , Smith SM . FSL. Neuroimage. 2012;62:782‐790. doi:10.1016/j.neuroimage.2011.09.015 21979382

[22] Basser PJ , Mattiello J , LeBihan D . MR diffusion tensor spectroscopy and imaging. Biophys J. 1994;66:259‐267. doi:10.1016/S0006-3495(94)80775-1 8130344 PMC1275686

[23] Yang F , Zhu YM , Kingsley PB . A further investigation of the Euler angle calculation in diffusion tensor imaging. 2016 IEEE 13th International Conference on Signal Processing (ICSP). IEEE; 2016:39‐43. doi. 10.1109/ICSP.2016.7877792

[24] Avants B , Tustison NJ , Song G . Advanced normalization tools: V1.0. The Insight Journal. 2009;2:1‐35. doi:10.54294/uvnhin

[25] Tournier JD , Smith R , Raffelt D , et al. MRtrix3: a fast, flexible and open software framework for medical image processing and visualisation. Neuroimage. 2019;202:116137. doi:10.1016/j.neuroimage.2019.116137 31473352

[26] Garyfallidis E , Brett M , Amirbekian B , et al. Dipy, a library for the analysis of diffusion MRI data. Front Neuroinform. 2014;8:8. doi:10.3389/fninf.2014.00008 24600385 PMC3931231

[27] Mori S , Oishi K , Jiang H , et al. Stereotaxic white matter atlas based on diffusion tensor imaging in an ICBM template. Neuroimage. 2008;40:570‐582. doi:10.1016/j.neuroimage.2007.12.035 18255316 PMC2478641

[28] Hua K , Zhang J , Wakana S , et al. Tract probability maps in stereotaxic spaces: analyses of white matter anatomy and tract‐specific quantification. Neuroimage. 2008;39:336‐347. doi:10.1016/j.neuroimage.2007.07.053 17931890 PMC2724595

[29] Wakana S , Caprihan A , Panzenboeck MM , et al. Reproducibility of quantitative tractography methods applied to cerebral white matter. Neuroimage. 2007;36:630‐644. doi:10.1016/j.neuroimage.2007.02.049 17481925 PMC2350213

