# Supplementary material for: Automatic determination of glymphatic flow with the DTI‐ALPS‐index along the principal axis system in native imaging space corrects for head and fiber orientation
Source: Magn Reson Med. 2025 Oct 5;95(2):1110–22. doi: 10.1002/mrm.70082 (PMC12681304; doi:10.1002/mrm.70082)
Supplement: Supplementary file 1 — Figure S1. (A) In addition to Figure 3.1, 3.2, the relation of ALPS‐LAB to ALPS‐PAS for multiple Cardan‐angle combinations with a simultaneous change of all angles is plotted. (B) Similar to (A) but setting two Cardan‐angles to zero while varying the third in the entire Cardan‐angle range. Figure S2. The curves represent the mean and standard deviation values of the three absolute angles computed for each voxel within the ROIs, which are defined per ALPS‐fiber‐region. For each angle, the mean and standard deviation were calculated across voxels within each ROI for each subject. Subsequently, these values were averaged across subjects. The results shown are for the right hemisphere as an illustration. The colors blue, green and red characterize (α,β,γ) in DWI space (Left: Manual ROIs in DWI space; Right: MNI ROIs in DWI space) and light blue, light green and light purple characterize (α,β,γ) in MNI space (MNI ROIs). Figure S3. A 3D RGB‐encoded map of diffusion images with manually (red, ROI‐option B) and automatically (white, ROI‐option C) placed ROIs in the native imaging space (DWI space) for subject one. Figure S4. Exemplary RGB maps in MNI space (upper) with MNI ROIs (white squares ROI‐option A) and in DWI space (down) with Manual ROIs in DWI space (red squares ROI‐option B) and MNI ROIs in DWI space (white squares ROI‐option C) of subject one, with (right) and without (left) head rotation. In all cases, the algorithm placed the ROIs (ROI‐option C) in anatomically meaningful areas (ALPS‐fiber‐regions green and blue areas). Note the smoothing effect of transforming the data to the MNI space. [file MRM-95-1110-s001.docx]

# Supporting information


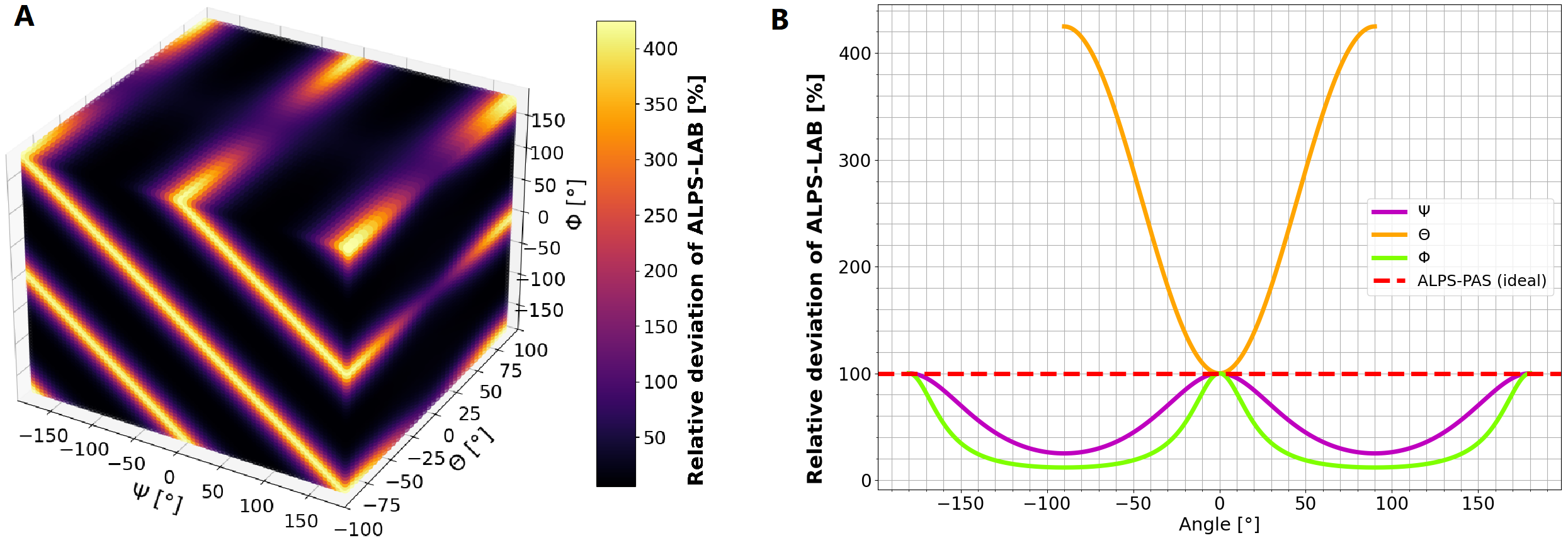


Supporting Figure S1: (A) In addition to Figure 3.1, 3.2, the relation of ALPS-LAB to ALPS-PAS for multiple Cardan-angle combinations with a simultaneous change of all angles is plotted. (B) Similar to (A) but setting two Cardan-angles to zero while varying the third in the entire Cardan-angle range.


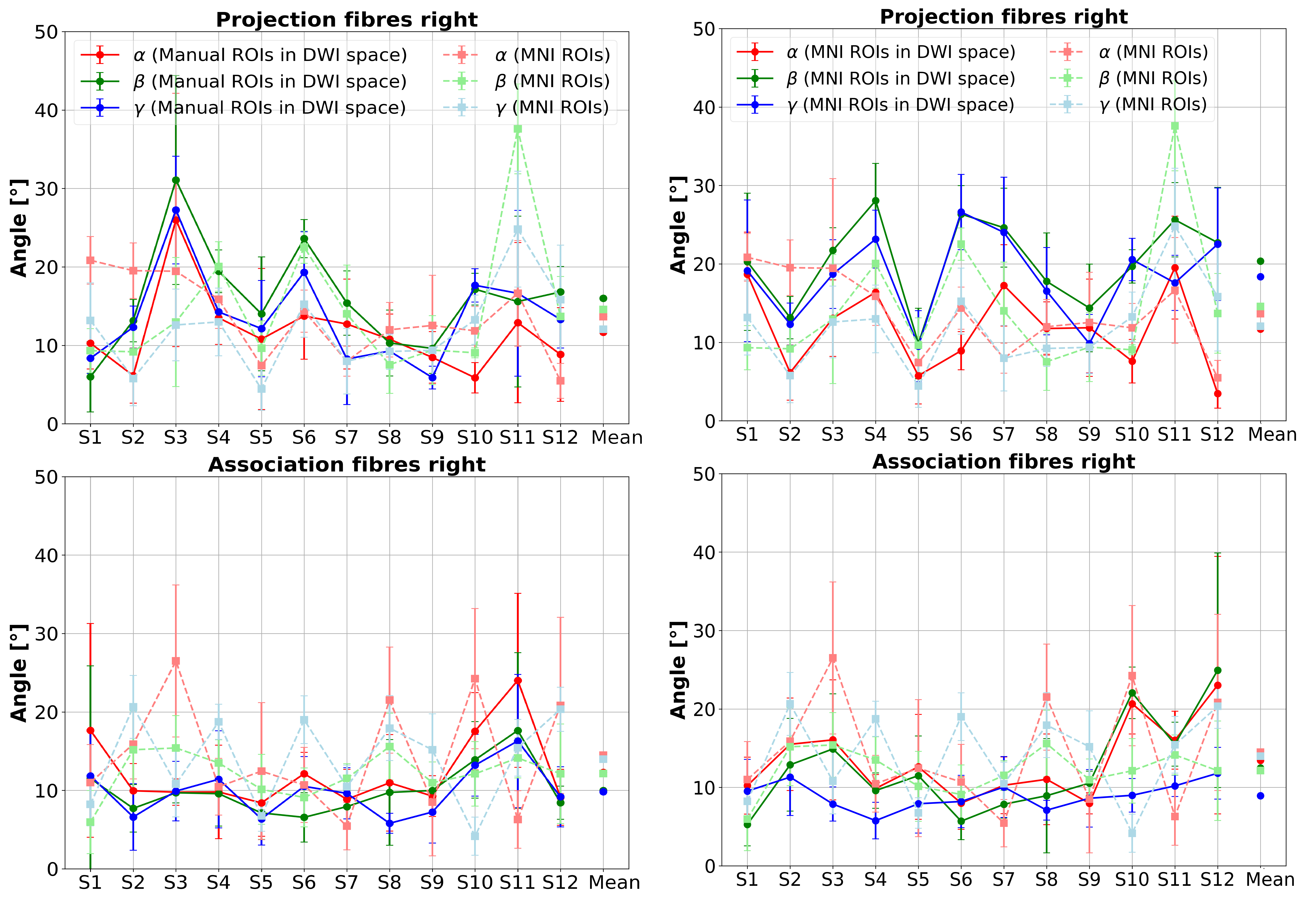


Supporting Figure S2: The curves represent the mean and standard deviation values of the three absolute angles computed for each voxel within the ROIs, which are defined per ALPS-fibre-region. For each angle, the mean and standard deviation were calculated across voxels within each ROI for each subject. Subsequently, these values were averaged across subjects. The results shown are for the right hemisphere as an illustration. The colours blue, green and red characterise $\left( \boldsymbol{\alpha, \beta, \gamma} \right)$ in DWI space (Left: Manual ROIs in DWI space; Right: MNI ROIs in DWI space) and light blue, light green and light purple characterise $\left( \boldsymbol{\alpha, \beta, \gamma} \right)$ in MNI space (MNI ROIs).


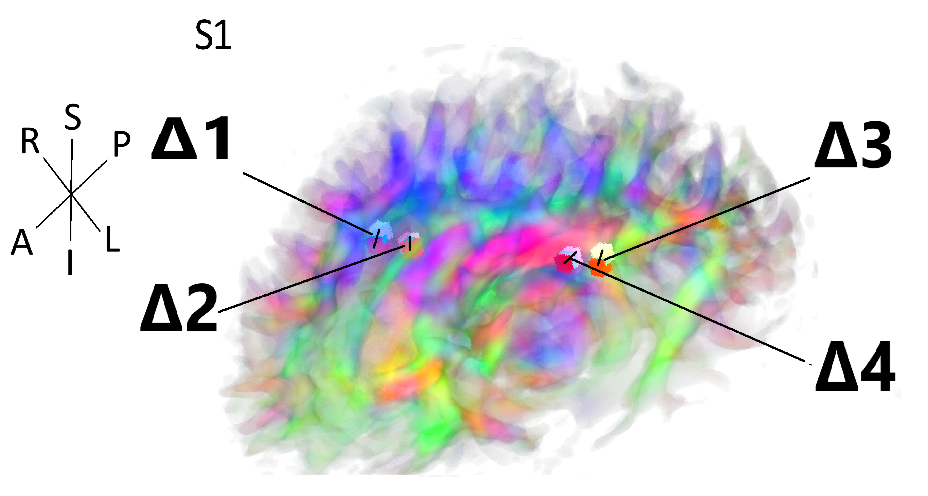


**Supporting Figure S3**: A 3D RGB-encoded map of diffusion images with manually (red, ROI-option B) and automatically (white, ROI-option C) placed ROIs in the native imaging space (DWI space) for subject one.


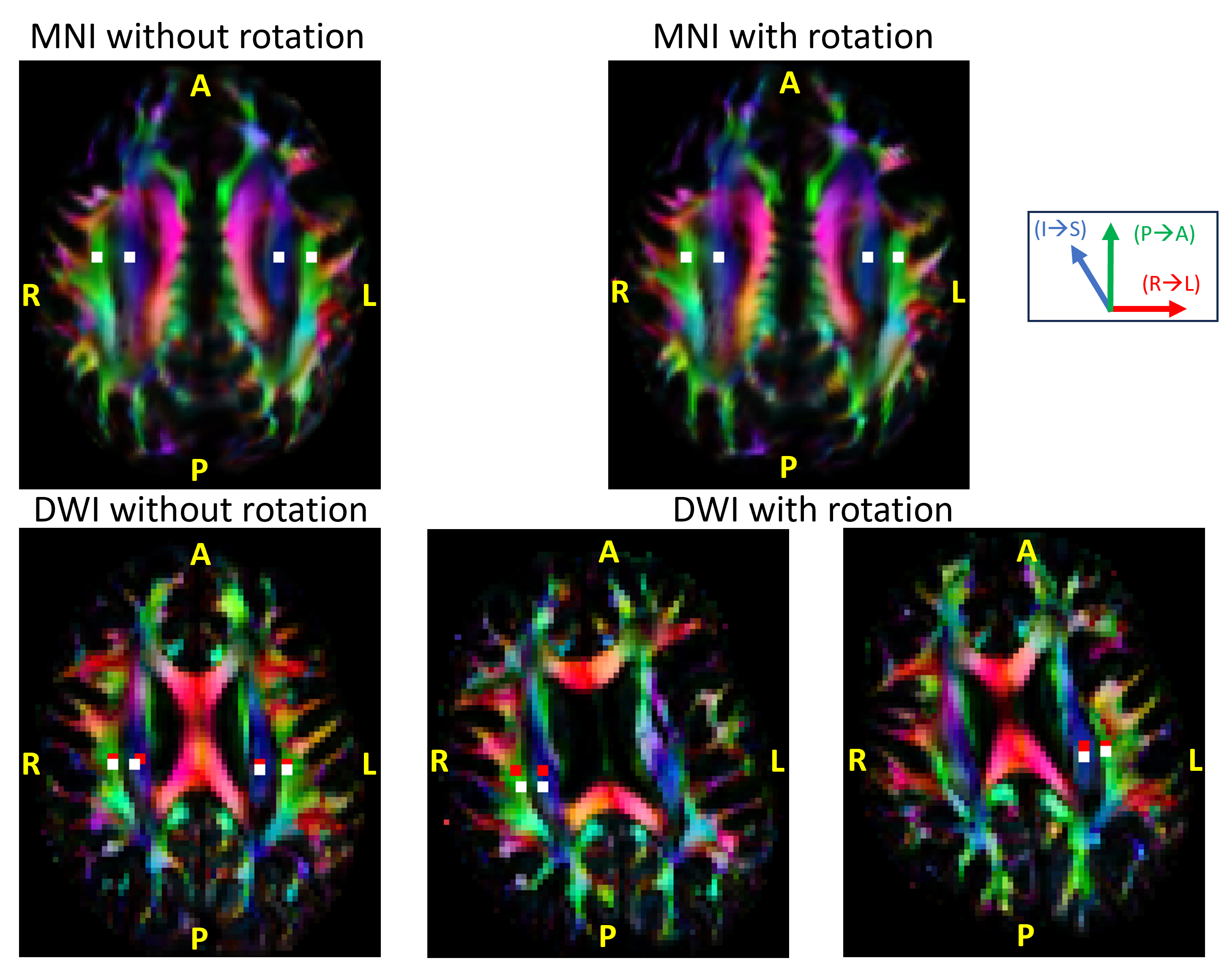


Supporting Figure S4: Exemplary RGB maps in MNI space (upper) with MNI ROIs (white squares ROI-option A) and in DWI space (down) with Manual ROIs in DWI space (red squares ROI-option B) and MNI ROIs in DWI space (white squares ROI-option C) of subject one, with (right) and without (left) head rotation. In all cases, the algorithm placed the ROIs (ROI-option C) in anatomically meaningful areas (ALPS-fibre-regions green and blue areas). Note the smoothing effect of transforming the data to the MNI space.
